# Hydrological Conditions Affect the Interspecific Interaction between Two Emergent Wetland Species

**DOI:** 10.3389/fpls.2017.02253

**Published:** 2018-01-15

**Authors:** Jian Zhou, Li-Di Zheng, Xu Pan, Wei Li, Xiao-Ming Kang, Jing Li, Yu Ning, Ming-Xiang Zhang, Li-Juan Cui

**Affiliations:** ^1^Institute of Wetland Research, Chinese Academy of Forestry, Beijing, China; ^2^Beijing Key Laboratory of Wetland Services and Restoration, Beijing, China; ^3^Beijing Hanshiqiao National Wetland Ecosystem Research Station, Beijing, China; ^4^School of Nature Conservation, Beijing Forestry University, Beijing, China

**Keywords:** hydrological fluctuation, water depth, interspecific interaction, *Scirpus planiculumis*, *Phragmites australis*, relative interaction index (RII)

## Abstract

Hydrological conditions determine the distribution of plant species in wetlands, where conditions such as water depth and hydrological fluctuations are expected to affect the interspecific interactions among emergent wetland species. To test such effects, we conducted a greenhouse experiment with three treatment categories, interspecific interaction (mixed culture or monoculture), water depth (10 or 30 cm depth), and hydrological fluctuation (static or fluctuating water level), and two common emergent wetland plant species, *Scirpus planiculumis* Fr. (Cyperaceae) and *Phragmites australis* var. *baiyangdiansis* (Gramineae). An increase in the water depth significantly restrained the growth of both *S. planiculumis* and *P. australis*, while hydrological fluctuations did not obviously alter the growth of either species. In addition, both water depth and hydrological fluctuations significantly affected the interspecific interaction between these two wetland species. *P. australis* benefited from interspecific interaction under increasing water depth and hydrological fluctuations, and the RII values were clearly positive for plants grown at a water depth that fluctuated around 30 cm. The results may have some implications for understanding how *S. planiculumis* and *P. australis*, as well as wetland communities, respond to the natural variation or human modification of hydrological conditions.

## Introduction

Plants in natural wetland ecosystems often experience hydrological disturbances (McGowan et al., 2011), and water level regulation is considered to be a useful tool in vegetation restoration (Leira and Cantonati, 2008; Yuan et al., 2017). Climate change causes strong hydrological disturbances, such as tidal variation, flooding, and extreme drought, which inevitably impose stress on plant individuals and communities, and numerous studies have examined the effects of such hydrological disturbances on the performance of plants in various transitional terrestrial and amphibious ecosystems (Wright et al., 2015; Wang and Li, 2016).

Abiotic disturbances and biotic interactions are important factors affecting plant community structure (Tilman, 1988; Grant et al., 2014). One classic approach for determining the spatial distribution of a plant community is identifying how ecological processes differ across environmental gradients (Keddy et al., 1997; Guo and Pennings, 2012). In recent decades, numerous studies have focused on plant-plant interactions along gradients of environmental disturbance (Bertness, 1991; Weigelt et al., 2002; Wang and Li, 2016) and have put forward the stress-gradient hypothesis (SGH), which predicts that facilitation and competition simultaneously affect neighboring plants and that the net outcome of these interactions shifts from negative to positive with increases in environmental stress (Bertness and Callaway, 1994; Maestre et al., 2009; Smit et al., 2009; He et al., 2013). This is a well-supported hypothesis (Liancourt et al., 2005; Maestre et al., 2005; Lortie and Callaway, 2006), but few studies have focused on the effects of hydrological disturbances, such as changes in water depth and hydrological fluctuations, on interspecific interactions among emergent wetland species (Luo et al., 2015).

In aquatic ecosystems, water depths fluctuate rather than being maintained at constant levels (Deegan et al., 2007; Wang P. et al., 2016). Increasing fluctuation and water depth can increase nutrient loss and tissue damage and decrease photosynthesis (Sasikala et al., 2008), which may cause species interactions to shift between facilitation and competition. There are also some opposing views, e.g., Bornette et al. (1994) found that aquatic plants performed best when flooding events occurred at an intermediate frequency, and Nielsen et al. (2013) found that increased hydrologic stability led to a loss of diversity in wetland communities. Because wetland species may differ in their responses and tolerance to hydrological changes, we predicted that water depth and hydrological fluctuations may greatly affect plant growth and interspecific interactions.

To better understand the importance of water depth and its fluctuations in the interactions among emergent plants in wetlands, we addressed the following questions: (1) Do water depth and hydrological fluctuations affect the growth of wetland plants? (2) Are the interspecific interactions among emergent species affected by hydrological fluctuations and water depth? To answer these questions, we conducted a greenhouse experiment testing the effects of three treatment categories (interspecific interactions, water depth, and hydrological fluctuations) on two common emergent wetland plants collected from the banks of the Chaobai River in Beijing.

## Materials and methods

### Species and sampling

We chose *Scirpus planiculumis* Fr. (Cyperaceae) and *Phragmites australis* var. *baiyangdiansis* (Gramineae) as the experimental species. Both species are cosmopolitan common plants that can create large stands and coexist in estuary wetlands (Thevs et al., 2007). *S. planiculumis* and *P. australis* are herbaceous clonal plants that reproduce both from seeds and from clonal stems or tubers in natural habitats (Peng et al., 2013; Dolinar et al., 2016). The mature size for *S. planiculumis* is 0.6–1 m and for *P. australis* is over 1 m (Chinese Wetland Vegetation's Commission., 1999). Hydrological conditions, including water depth, hydrological fluctuation, and water temperature and so on, is a key factor for the survival and spread of both species (Dolinar et al., 2016). In addition, *P. australis* is considered to be a malignant invasive species in North America because of its strong stress resistance ability (Kowalski et al., 2015).

We collected young ramets of similar size of both *S. planiculumis* and *P. australis* from a single location on the north bank of the Chaobai River, Shunyi District, Beijing (40°06′55.6″ N, 116°43′3.5″ E) in early April 2016. As observed, these two species coexist in nature during the beginning of the growing season. Over 160 ramets per species were collected and grown in a greenhouse (located at the Wildlife Rescue & Rehabilitation Center, Shunyi District, Beijing, China) for 10 days in tap water to acclimate them to indoor conditions before exposing them to the experimental hydrological treatments. We discarded plants that exhibited any transplanting stress during these 10 days and then collected 72 single thriving ramets of both *S. planiculumis* and *P. australis* for the experiment. We measured the initial average total dry mass and the average height of both species before transplanting; the initial measurements were 3.11 ± 0.73 g and 16.32 ± 3.22 cm, respectively, for the ramets of *S. planiculumis* and 2.14 ± 0.82 g and 14.31 ± 6.14 cm, respectively, for the ramets of *P. australis*.

### Experimental design

**Figure 1:**
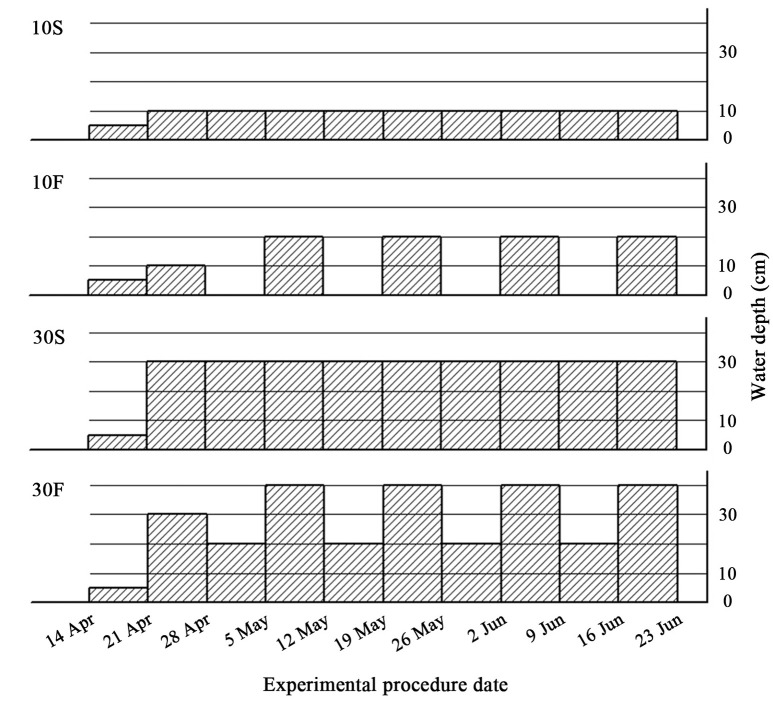
Experimental design. Schematic representation of the four hydrological treatments. 10S, static water level at 10 cm; 10F, water depth fluctuating from 10 cm with a 10 cm amplitude; 30S, static water level at 30 cm; 30F, water depth fluctuating from 30 cm with a 10 cm amplitude. The shaded areas indicate the water depth, and abscissae indicate the date of the experiment in 2016. The first two weeks, from 14 April to 28 April, constituted a pretreatment stage, and the treatments lasted for 8 weeks, until 23 June 2016.

A standard replacement series experiment was conducted in this study (Gibson et al., 1999; Jolliffe, 2000). Plants were subjected to two interspecific interaction treatments crossed with four hydrological treatments. *S. planiculumis* and *P. australis* were planted as mixed cultures and monocultures at three different plant density ratios (0:4, 2:2, and 4:0). To simulate the fluctuations in water that naturally occur in estuary wetlands, we established four treatments: (1) static water level at 10 cm, coded as 10S; (2) water depth fluctuating from 10 cm with a 10 cm amplitude, which means that the water depth was alternately maintained at 0 and 20 cm over a cycle of 1 week, coded as 10F; (3) static water level at 30 cm, coded as 30F; and (4) water depth fluctuating from 30 cm with a 10 cm amplitude, which means that the water depth was alternately maintained at 20 and 40 cm over a 1-week cycle, coded as 30F (as shown in Figure 1). We set up six replicates for each treatment, and there were 72 plastic containers (27.5 × 27.5 × 15 cm deep) in total. We divided these containers into four groups (18 containers per group) and placed them into four concrete tanks (190 cm length × 90 cm width × 80 cm deep); one previously described hydrological treatment was established in each tank.

On 14 April 2016, four single ramets of the two species were transplanted vertically into each plastic container. The containers were filled to 10 cm with a substrate composed of two soil types in a 1:1 (v/v) ratio: one was collected from the bank of an artificial lake located at the Beijing Wildlife Rescue & Rehabilitation Center, and the other was sand purchased from a construction company. The mixed substrate contained 0.31 (0.01) mg total N [mean (SE); *N* = 3], 0.55 (0.03) mg total P, 1.33 (0.09) mg K, and 8.66 (0.71) mg organic matter g^−1^ dry mass of soil based on an analysis performed in a lab at the Institute of Botany, Chinese Academy of Sciences, in Beijing.

After allowing the plants to establish for 1 week, on 21 April 2016, we added tap water to 10 or 30 cm and kept water levels static for another week to ensure the survival of all plants during the experimental period. On 28 April 2016, we began to alter the water level to simulate natural water fluctuations (as shown in Figure 1). We added tap water to the 10S and 30S tanks each week during the experiment to maintain the water levels. The mean temperature was 19.2°C in the greenhouse during the experiment.

All the *S. planiculumis* and *P. australis* plants were harvested after 8 weeks, on 23 June 2016. We measured the total height and counted the number of ramets of both species in each pot. All the plants were separated into above- and belowground parts, oven dried at 70°C for 72 h and weighed.

### Data analysis

We analyzed the growth data for *S. planiculumis* and *P. australis* separately. We used two-way ANOVA to test the effects of interspecific interactions (mixed culture and monoculture) and hydrological conditions (10S, 10F, 30S, and 30F) on growth and morphological variables (including total biomass, above- and belowground biomass, number of ramets, average height per ramet, and root-to-shoot ratio) for both species. Following the two-way ANOVA, Dunnett's test was used to compare the means of the growth data among the four hydrological conditions while accounting for each of the four treatments. To test the effects of interspecific competition, water depth, and hydrological fluctuations on the growth and morphological data for *S. planiculumis* and *P. australis*, we conducted three-way ANOVA. Significant differences were identified using *post-hoc* Tukey's honest significant difference tests. Mean values per species in a container were used in these analyses. Some data were square root transformed before analysis to meet the requirements of homoscedasticity and normality; these data are indicated in the tables.

We analyzed the competitive responses of *S. planiculumis* and *P. australis* using the relative interaction index (RII) based on the total biomass data. The RII is suitable for evaluating positive and negative interactions between plants because it can compare the performance of each species when grown in mixed culture to its performance in monoculture and can also effectively measure the competition intensity between two species (Weigelt and Jolliffe, 2003; Armas and Pugnaire, 2004). The RII is calculated using the following formulae:

$$RII_{a}=(Y_{ab}-Y_{a})/(Y_{ab}+Y_{a})$$

$$RII_{b}=(Y_{ba}-Y_{b})/(Y_{ba}+Y_{b})$$

where *Y* is the total biomass per plant in each experimental container, a and b represent the two species separately, *Y*_*a*_ is the total biomass of species a when grown alone, and *Y*_*b*_ is the total biomass of species b when grown alone. *Y*_*ab*_ is the total biomass of species a when grown with species b, and *Y*_*ba*_ is the total biomass of species b when grown with species a. RII = 0 indicates that there is no significant difference between the effects of the mixed culture and monoculture treatments on plant growth, while positive values indicate that the interaction is facilitative, and negative values indicate that the interaction is competitive. Finally, one-way ANOVA was used to test the effects of the hydrological conditions on the RII. All the analyses were conducted using SPSS 20.0 (SPSS, Chicago, IL, USA).

## Results

### Effects of hydrological conditions on the growth of *Scirpus planiculumis*

**Figure 2:**
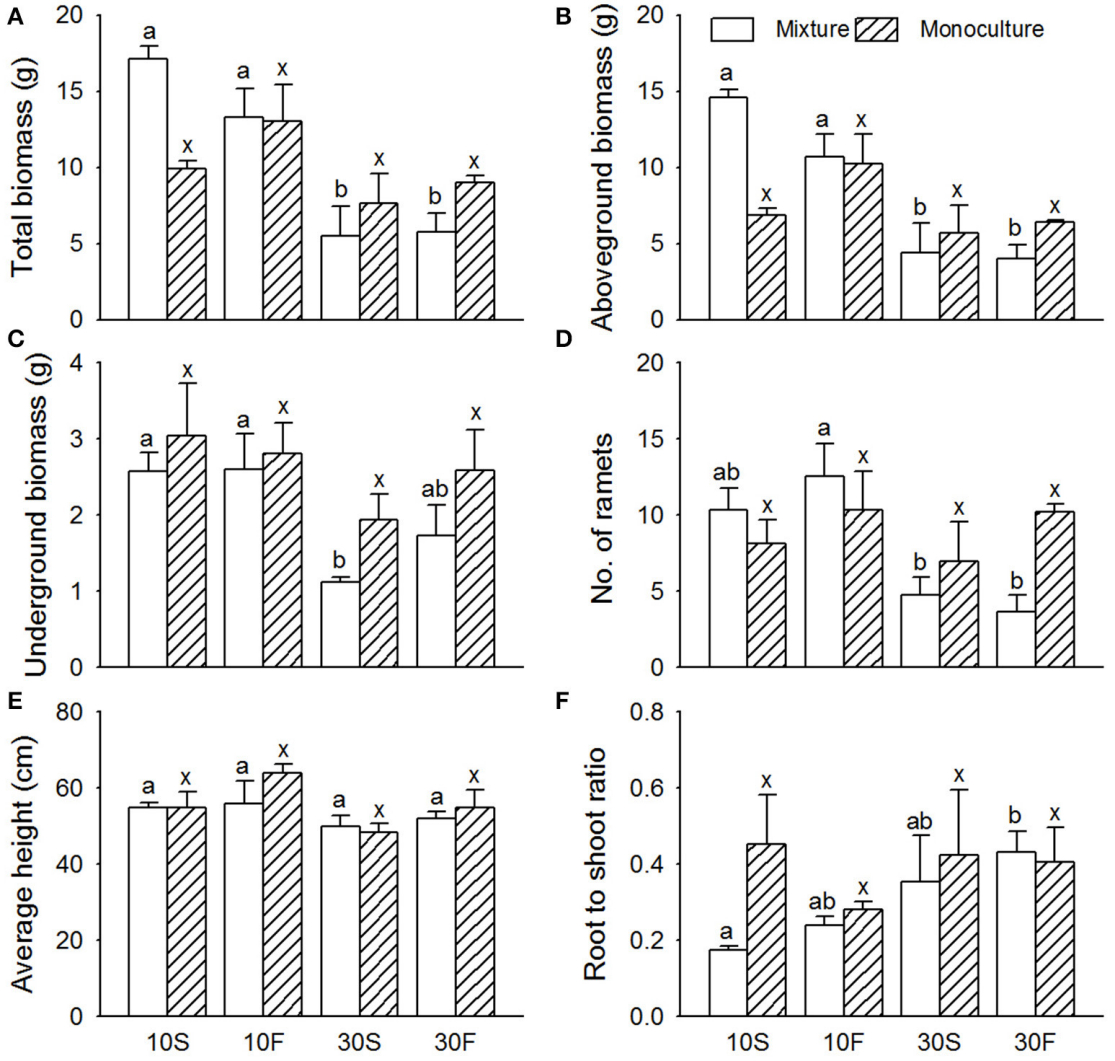
Effects of interspecific interaction and hydrological conditions on the **(A)** total biomass, **(B)** aboveground biomass, **(C)** belowground biomass, **(D)** number of ramets, **(E)** average height, and **(F)** root-to-shoot ratio (mean + SE) of *Scirpus planiculumis*. Different letters indicate a significant difference among mixture or monoculture treatments (at *P* < 0.05).

As expected, the different hydrological conditions affected all the growth data for *S. planiculumis* except the root-to-shoot ratio (*P* = 0.362 in Table 1; Figure 2). Specifically, water depth had a greater impact than hydrological fluctuation on the growth of *S. planiculumis* (Table 2); the hydrological fluctuation treatment affected only the average height per plant (*P* = 0.078; Table 2).

**Table 1:** Two-way ANOVA results for the effects of interspecific interaction (with or without interspecific interactions) and four hydrological conditions (including 10S, 10F, 30S, and 30F treatments) on growth and morphological data for *Scirpus planiculumis* and *Phragmites australis*.

	**Interspecific interaction (I)**		**Hydrological conditions (H)**		**I** × **H**	
	***F*_(1, 16)_**	***P***	***F*_(3, 16)_**	***P***	***F*_(3, 16)_**	***P***
***SCIRPUS PLANICULUMIS***						
Total biomass^*^	0.22	0.644	**11.29**	<**0.001**	**4.52**	**0.018**
Aboveground biomass^*^	1.35	0.262	**11.02**	<**0.001**	**5.64**	**0.008**
Belowground biomass	3.77	0.070	**3.73**	**0.033**	0.26	0.855
No. of ramets	0.80	0.385	**4.04**	**0.026**	2.90	0.067
Average height	0.90	0.358	3.22	0.051	0.74	0.544
Root-to-shoot ratio	1.82	0.196	1.14	0.362	0.96	0.437
***PHRAGMITES AUSTRALIS***						
Total biomass	1.04	0.323	**6.57**	**0.004**	**4.30**	**0.021**
Aboveground biomass^*^	0.70	0.415	**6.85**	**0.004**	**4.87**	**0.014**
Belowground biomass	1.70	0.211	**3.58**	**0.038**	1.45	0.265
No. of ramets	**4.95**	**0.041**	**3.35**	**0.045**	1.79	0.190
Average height	0.35	0.561	1.08	0.386	0.94	0.447
Root-to-shoot ratio	0.28	0.603	2.34	0.112	0.69	0.572

* *Indicates these data were transformed to meet the requirements of homoscedasticity and normality*.

*Bold text indicates a significant difference (P < 0.05)*.

**Table 2:** Three-way ANOVA results for the effects of interspecific interaction (with or without interspecific interactions), water depth (10 or 30 cm water depth), and hydrological fluctuations (static or fluctuant water level) on the growth and morphological data for *Scirpus planiculumis* and *Phragmites australis*.

	**Interspecific interaction (I)**	**Water depth (W)**	**Hydrological fluctuations (F)**	**I × W**	**I × F**	**W × F**	**I × W × F**
***SCIRPUS PLANICULUMIS***							
Total biomass	0.222^ns^	33.556^***^	0.042^ns^	8.450^*^	3.389^ns^	0.273^ns^	1.717^ns^
Aboveground biomass	1.352^ns^	32.999^***^	0.002^ns^	9.612^**^	4.778^*^	0.052^ns^	2.542^ns^
Belowground biomass	3.771^ns^	9.051^**^	0.757^ns^	0.679^ns^	0.031^ns^	1.393^ns^	0.062^ns^
No. of ramets	0.796^ns^	10.128^**^	1.769^ns^	7.168^*^	0.737^ns^	0.207^ns^	0.796^ns^
Average height	0.897^ns^	6.118^*^	3.541^ns^	0.477^ns^	1.620^ns^	0.013^ns^	0.119^ns^
Root-to-shoot ratio	1.821^ns^	3.026^ns^	0.032^ns^	1.049^ns^	1.557^ns^	0.369^ns^	0.265^ns^
***PHRAGMITES AUSTRALIS***							
Total biomass	1.038^ns^	16.809^**^	2.551^ns^	8.401^*^	4.136^ns^	0.342^ns^	0.355^ns^
Aboveground biomass	0.699^ns^	18.626^**^	1.629^ns^	9.797^**^	4.628^*^	0.297^ns^	0.192^ns^
Belowground biomass	1.695^ns^	5.814^*^	4.596^*^	2.162^ns^	1.358^ns^	0.317^ns^	0.833^ns^
No. of ramets	4.949^*^	8.849^**^	1.189^ns^	4.347^ns^	0.871^ns^	0.009^ns^	0.141^ns^
Average height	0.352^ns^	1.920^ns^	0.001^ns^	0.314^ns^	1.529^ns^	1.315^ns^	0.960^ns^
Root-to-shoot ratio	0.281^ns^	6.706^*^	0.308^ns^	1.035^ns^	0.024^ns^	0.001^ns^	1.010^ns^

F-values and significance levels (

*** *P < 0.001*,

** *0.001 < P < 0.01*,

* *0.01 < P < 0.05) are given*.

The effects of water depth on the total biomass, aboveground biomass, and number of ramets varied significantly between the monoculture and mixed culture treatments (Table 2; significant effect of I × W): (1) plants performed much better in mixed culture than in monoculture at the 10 cm water depth, and (2) we obtained the opposite result for the 30 cm water depth (Figure 2). Moreover, disturbances caused by hydrological fluctuations also changed the effect of interspecific interactions for *S. planiculumis* (Table 2; significant interaction effect of I × F on the total biomass and aboveground biomass): aboveground biomass (*P* = 0.044) of plants significantly increased in mixed culture compared with these biomass values in the monoculture treatment under static hydrological conditions, while these differences were not obvious under the fluctuating conditions (Figure 2).

### Effects of hydrological conditions on the growth of *Phragmites australis*

**Figure 3:**
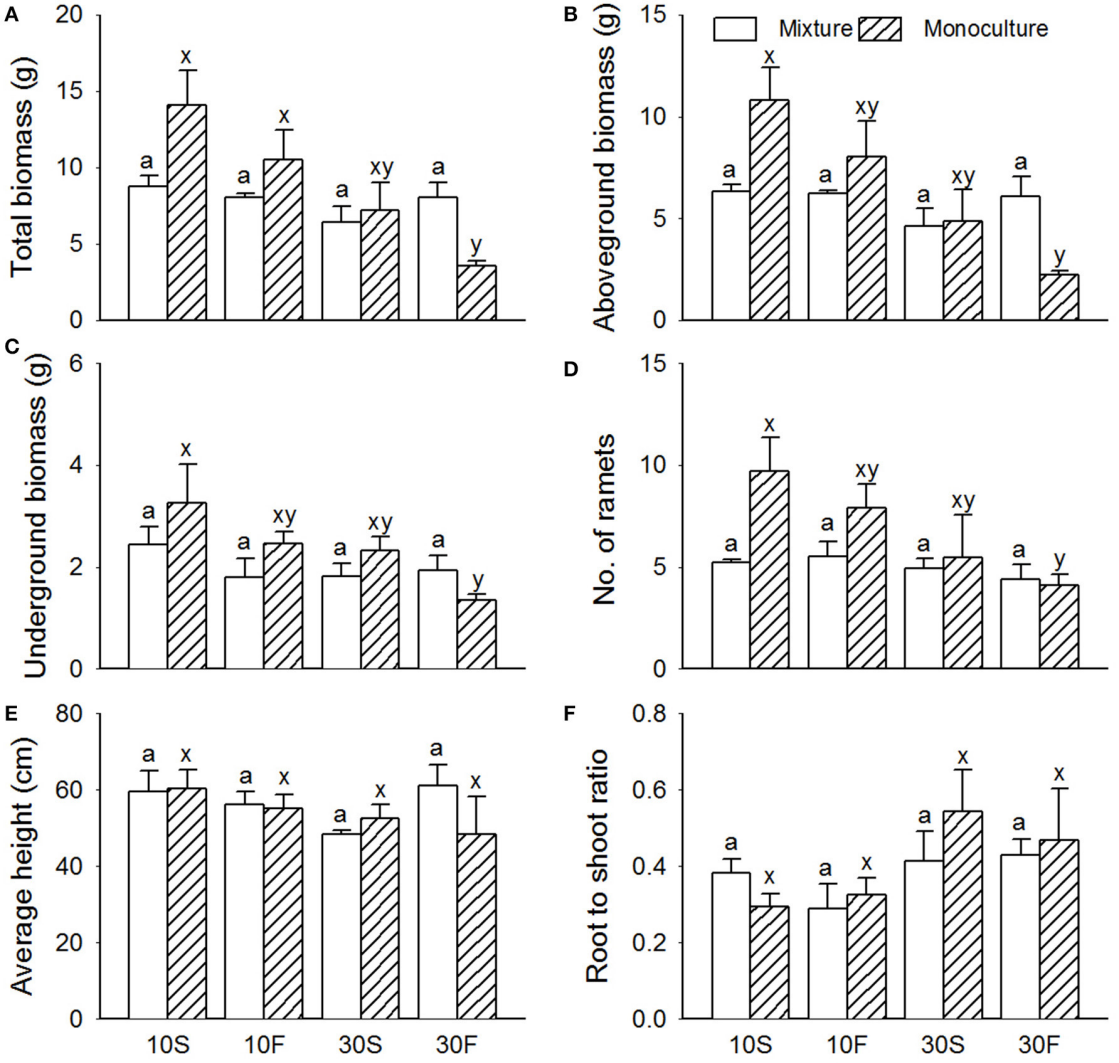
Effects of interspecific interaction and hydrological conditions on the **(A)** total biomass, **(B)** aboveground biomass, **(C)** belowground biomass, **(D)** number of ramets, **(E)** average height, and **(F)** root-to-shoot ratio (mean + SE) of *Phragmites australis*. Different letters indicate a significant difference among mixture or monoculture treatments (at *P* < 0.05).

Similar to the results for *S. planiculumis*, the total biomass, aboveground biomass, belowground biomass and number of nodes in *P. australis* decreased with increased disturbance caused by fluctuating hydrological conditions (Table 1; Figure 3). The number of nodes was 30% higher in the monoculture than in the mixed culture treatment (Figure 3). According to the three-way ANOVA, an increase in water depth decreased all the growth variables for *P. australis* except average height, while hydrological fluctuations did not alter the growth of *P. australis* (Table 2; Figure 3) with the exception of the belowground biomass (*P* = 0.048; Table 2).

The interactive effects of interspecific interaction and water depth were obvious (Table 2): mixed cultures with *S. planiculumis* significantly decreased the total and aboveground biomass of *P. australis* by nearly 30% in the 10 cm water depth treatment, but these effects disappeared in the 30 cm water depth treatment (Figures 3A,B). The interactive effects of interspecific interaction and hydrological fluctuation also affected the total and aboveground biomass of *P. australis* (Table 2). There were no other interactive effects.

### Results for RII

**Figure 4:**
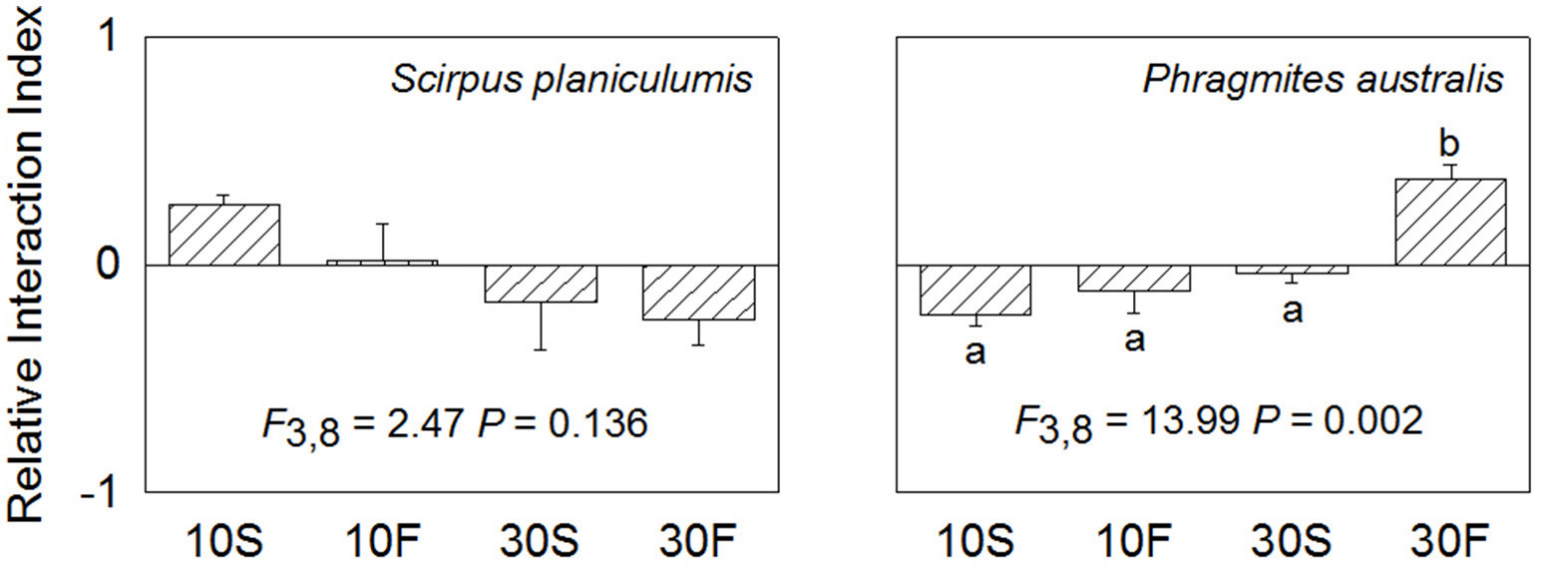
Relative interaction index (RII) of *Scirpus planiculumis* and *Phragmites australis* in monoculture or mixed culture under four hydrological conditions. Shown are means ± SE. Different letters indicate a significant difference among treatments (one-way ANOVA followed by Dunnett's test).

The average RII values for *S. planiculumis* were positive when grown at the 10 cm water depth and were negative at the 30 cm water depth; however, there were no significant differences among the four RII values for this species (*P* = 0.136). The mean RII values for *P. australis* increased with increasing water depth and fluctuation; the value in the 30F treatment was obviously positive and significantly different from the other three values (*P* = 0.002; Figure 4).

## Discussion

The growth of both species was significantly decreased at the 30 cm water depth (Table 1), agreeing with the results of many previous studies (Voesenek et al., 2006; Panda et al., 2008). The 30 cm water depth was much higher than the initial height of both *S. planiculumis* and *P. australis*, which may cause reductions in the oxygen supply and carbohydrate use efficiency and increases in anaerobic respiration and nutrient consumption (Gibbs and Greenway, 2003), leading to an overall decrease in biomass accumulation. In nature, adult *P. australis* can survive in relatively deep water, over 1 m, because the mother ramets can provide nutrients to water-stressed seedlings through clonal integration. In our study, all the plants of both species were single ramets at the beginning, and the initial height was markedly lower than the static water level of 30 cm and higher than 10 cm. *P. australis* gained relatively more biomass in the 10 cm treatments than in the so cm treatments at the beginning of growth, and we predicted than the 30 cm water level will benefit the *P. australis* in later stages. Furthermore, many emergent species can morphologically adapt to submergence stress by elongating their shoots or leaves (Wang et al., 2014). In the case of *S. planiculumis*, the average height increased markedly with increasing water depth (Table 2), demonstrating morphological adaptation. Thus, morphological adaptation may explain the response of emergent species to water depth.

Some studies have found that hydrological fluctuations cause plants to become re-exposed to the ambient oxygen environment, resulting in the production of acetaldehyde and reactive oxygen species (Boamfa et al., 2005; Luo et al., 2012; Steffens et al., 2013), which are harmful to plant growth. However, the hydrological fluctuations did not obviously alter the growth of the two study species (Table 2). Some previous studies have found that plants are able to tolerate fluctuations of up to 30 cm without a significant drop in final biomass (Deegan et al., 2007; Wang P. et al., 2016). Luo et al. (2015) found that a small amount of hydrological fluctuation did not alter the growth of wetland communities because small changes may allow parts of the plant shoots to remain in the water, which causes the production of fewer oxidative toxins that can injure the plant tissue. In our study, the 20 cm range of fluctuation may have been too small to affect the growth of these two emergent species. Another possible explanation for this result may be that both *S. planiculumis* and *P. australis* have a strong ability to tolerate such water fluctuations and exhibit compensatory growth. Previous studies have also found that some other sources of stress or resources, such as low soil nutrients, nitrogen pulses, and altered water content (Geng et al., 2006; Sun et al., 2009; Wang et al., 2015), could not change the growth of target plants because the plants were insensitive to such stresses.

The inhibition or promotion of growth may be caused not only by submergence stress but also by interspecific interactions with neighbors (Wang Y. J. et al., 2016), and both interspecific and intraspecific interactions can be affected by the hydrological conditions. There is a trade-off between facilitation and competition in the interactions among plants, which means that plants exhibit greater competition in a suitable environment and more facilitation in a stressful environment (Maestre et al., 2009; Zhou et al., 2017).

Both the hydrological fluctuation and water depth treatments significantly affected the interspecific interactions between *S. planiculumis* and *P. australis* (Table 2; Figures 1, 2). Specifically, the mean RII values for *P. australis* increased with increasing water depth and fluctuation, while the values for *S. planiculumis* decreased (Figure 4). *P. australis* benefited more from the interspecific interaction than *S. planiculumis* when disturbance caused by fluctuating hydrological conditions increased; the plants performed much better in mixed culture than in monoculture under the fluctuating water conditions at 30 cm. This result mainly occurred because different plant species exhibit different levels of environmental tolerance, and *P. australis* appears to be better able to adapt to greater water depths and increasing fluctuation, such as those evaluated here, than *S. planiculumis*. For instance, Edwards et al. (2003) found that the amount of time necessary for morphological adaptation will depend on many factors, but, as an indication, it took the macrophyte *Eleocharis cellulosa* 3 weeks to adjust its morphological characters to new water level conditions and 9 weeks to shift its biomass partitioning. In addition, Deegan et al. (2007) established hydrological fluctuation treatments for emergent wetland species; *Triglochin procerum* did not respond to the treatments due to its ability to photosynthesise under water, while *Typha domingensis* responded negatively to increasing amplitude, and other species responded positively.

In nature, *S. planiculumis* and *P. australis* coexist during the early growing season (April to June). *P. australis* dominates the relatively deep-water areas through its clonal growth and morphological characters. Mauchamp and Mésleard (2001) found that the tolerance of *P. australis* to submergence stress increased with maturity, which indicates that time affects the interspecific interactions. Unlike *P. australis, S. planiculumis* accumulates more biomass under competition with its neighbors in a relatively static environment, but these effects disappear when hydrological disturbance is increased (Figure 2B). The results also indicate that *P. australis* is better able to adapt to disturbance than *S. planiculumis*. Some studies have found that the adaptive strategies of plants under high levels of disturbance caused by changing hydrological conditions mainly include (1) morphological adaptations via stem elongation and the formation of adventitious roots and aeration tissues, (2) alterations to metabolism, and (3) the regulation of physiological activity by changing the concentrations of hormones such as ethylene, gibberellin, and abscisic acid (Vasellati et al., 2001; Zhang et al., 2007).

## Conclusions

Our study showed that (1) both *S. planiculumis* and *P. australis* accumulated more biomass at a relatively low water level (10 cm) than at a high water level, which indicates that water depth and wetland emergent plant growth were negatively correlated during the early growing season and that hydrological fluctuations did not obviously alter the growth of either target species; and (2) *P. australis* benefited from its interspecific interactions with *S. planiculumis*, which shifted from competition to facilitation with increasing water depth and hydrological fluctuations. These results partly explain the distribution patterns of both *S. planiculumis* and *P. australis* in relation to different hydrological conditions. *P. australis* showed an advantage in using varied hydrological conditions in competition with other emergent species, such as *S. planiculumis*. Moreover, these results may also contribute to our understanding of the organization and assembly of estuarine wetland plant communities and may have important implications for understanding how emergent wetland plant communities respond to the natural variation and human modification of hydrological conditions. However, the lack of O_2_ content data and ORP data may limit the understanding and the results will vary in relation to different biotic and abiotic environmental stressors and their complex combinations in natural habitats. Further studies should evaluate the shifts between competition and facilitation in response to multiple environmental stressors in wetland plant communities.

## Author contributions

JZ and L-JC conceived and designed the experiments; JZ, L-DZ, XP, and YN executed the experiment and measured the data; JZ, L-DZ, and JL analyzed the data and made the figures; L-JC, WL, and X-MK contributed to writing and editing the manuscript; JZ wrote the paper; JZ, L-DZ and M-XZ revised the paper.

### Conflict of interest statement

The authors declare that the research was conducted in the absence of any commercial or financial relationships that could be construed as a potential conflict of interest.
